# The Overlap of Diabetes and Osteoarthritis in American Populations

**DOI:** 10.7759/cureus.38287

**Published:** 2023-04-29

**Authors:** Harshita Nadella, Allan W Bloom, Michelle Demory Beckler, Marc M Kesselman

**Affiliations:** 1 Rheumatology and Immunology, Nova Southeastern University Dr. Kiran C. Patel College of Osteopathic Medicine, Davie, USA; 2 Internal Medicine, Nova Southeastern University Dr. Kiran C. Patel College of Osteopathic Medicine, Davie, USA; 3 Microbiology and Immunology, Nova Southeastern University Dr. Kiran C. Patel College of Allopathic Medicine, Fort Lauderdale, USA; 4 Rheumatology, Nova Southeastern University Dr. Kiran C. Patel College of Osteopathic Medicine, Nova Southeastern University, Davie, USA

**Keywords:** oxidative stress, obesity, inflammatory cytokines, diabetes mellitus, osteoarthritis

## Abstract

Diabetes mellitus, a condition in which the body’s ability to produce insulin is impaired, and osteoarthritis (OA), a painful degeneration of joint cartilage, are both serious conditions that affect millions of people in the United States (U.S.). Osteoarthritis is a chronic degenerative condition of the joint cartilage, affecting mainly the older population. The purpose of this paper is to find a connection, if any, between diabetes and osteoarthritis and if either condition can predispose an individual to the other. Not only can this review help to explain the co-existence of these two diseases, but it can also be used to look into a cure for patients in the future. After preliminary searches were done on PubMed, results were narrowed using specific keywords and similar risk factors among the two diseases. It was found that these two conditions are actually interrelated due to oxidative stress and pro-inflammatory cytokines. Seeing the high risk of developing one of these conditions and that obesity, one of the biggest risk factors for both diabetes and osteoarthritis, is at an all-time high in this country, a possible connection between the two of these diseases is very prevalent to look into. This information can be used to help correlate not only a better-targeted treatment but also lead to future research into why obesity is one of the biggest risk factors for both conditions.

## Introduction and background

Introduction

Diabetes mellitus (diabetes) is a chronic condition that impairs the body’s ability to either produce or respond to the hormone insulin, which is responsible for regulating the body’s glucose levels [1]. Within the United States (U.S.) population, in 2021, there were an estimated 34.2 million people of all ages diagnosed with diabetes (types I and II), which represents 10.5% of the entire U.S. population [1]. Currently, in 2022, there is an additional nearly 3% of the US population is estimated to have undiagnosed diabetes type II due to a lack of regular medical examinations [1].

As evident in the table, diabetes is an extremely prevalent disease that affects all races, sexes, and ages (Table 1). It is important to note, however, that in type 1 diabetes, the body fails to make insulin due to the destruction of beta cells in the pancreas; this is more of a genetic disorder, so it is seen with a higher prevalence in younger populations. On the other hand, in type 2 diabetes, the body either develops resistance to insulin or not enough insulin is produced to lower the blood sugars; this is more often due to old age and insulin resistance. Although there is a strong correlation between increasing age and the onset of diabetes, diabetes type I affects younger people as well. Age is also another key common factor within the overlap of diabetes and osteoarthritis (OA). While diabetes affects both men and women, there is a slightly higher rate of diabetes in men than in women [2]. The prevalence of overall diagnosed diabetes was highest among American Indians and non-Hispanic blacks. Among those included in the Hispanic populations, Mexicans (14.4%) and Puerto Ricans (12.4%) had the highest prevalence, followed by Central/South Americans at 8.3% and Cubans at 6.5% [3].

**Table 1:** The estimated prevalence of diabetes in the U.S. (diagnosed, undiagnosed, and total) among ages 18 and older in 2016 [3]. Figure original © Harshita Nadella.

Characteristic	Diagnosed diabetes percentage	Undiagnosed diabetes percentage	Total diabetes percentage
Total	10.5%	2.8%	13.3%
Age			
18–44	3%	1.1%	4.2%
45–64	13.8%	3.6%	17.5%
>65	21.4%	5.4%	26.8%
Sex			
Men	11.0%	3.1%	14.0%
Women	9.5%	2.5%	12.0%
Race/ethnicity			
White, non-Hispanic	9.4%	2.5%	11.9%
Black, non-Hispanic	13.3%	3.0%	16.4%
Asian, non-Hispanic	11.2%	4.6%	14.9%
Hispanic	10.3%	3.5%	14.7%

Low levels of education are another key social determinant that seems to have a correlation with higher diabetes prevalence [4]. Education is an indicator of socioeconomic status. Specifically, 13.3% of adults with less than a high school education had been diagnosed with diabetes, versus 9.7% of those with a high school education and 7.5% of those with more than a high school education [4]. Rates of diagnosed diabetes have been increasing steadily since 1999. Since then, the rate of diagnosed diabetes has increased from 6% during that year to nearly 10% in 2022. Meanwhile, rates of undiagnosed diabetes remained constant at around 3% [4].

There are many key risk factors that are associated with diabetes, especially type 2 diabetes. Specifically, among those patients with type II diabetes, 21.6% were tobacco users based on various self-reporting measurements or levels of serum nicotine, and 36.4% had quit smoking but had a history of smoking at least 100 cigarettes in their lifetime [3,4]. Other key risk factors, similar to OA, are overweight and obesity. In addition, 89% of those with diagnosed diabetes were overweight or had an obesity (body mass index, BMI) of 25 kg/m^2^ or higher [3,4]. Adipokines, which are cell-signaling molecules produced by the adipose tissue in the body in order to regulate metabolic processes of the body, inflammatory and obesity in particular, have been a keen area of interest. As a result, adipokines are also found in higher numbers in individuals struggling with obesity and diabetes. In addition, physical inactivity (less than 10 minutes a week of physical activity) was also reported in 38% of the obese population. Hypertension, or high blood pressure, was also noted in 68.4% of the population, in which an individual presented with a systolic blood pressure of 140 mmHg or higher, a diastolic blood pressure of 90 mmHg or higher, or was on prescription medication for their high blood pressure [4]. Furthermore, high cholesterol levels (non-HDL levels of 130 mg/dL or higher) were noted in 43.5% of the population [4].

OA is defined as "degeneration of joint cartilage and the underlying bone, most common from middle age onward. It causes pain and stiffness, especially in the hip, knee, and thumb joints" [5]. OA commonly occurs among middle-aged to elderly individuals but can occur in younger generations of individuals, especially those with comorbid conditions. The most common symptoms or signs associated with OA include joint pain, stiffness, tenderness, and loss of flexibility [6]. OA is associated with loss of joint space as well as degeneration and loss of cartilage. Cartilage is a flexible connective tissue found between structures that acts as a cushion. When "wear and tear" causes it to break down, the underlying bone starts to become exposed and goes through a period of painful cellular degenerative changes [6].

There are more than 100 different types of arthritis, but OA remains the most common form, affecting 32.5 million adults in the U.S. [7]. It is projected to affect almost 78 million adults by the year 2040 [6]. Age is a significant factor in the burden of osteoarthritis, with 88% of those with OA being 45 years of age or older [7]. OA is also first detected in a patient with the highest occurrence in the age group 55-64 [7]. OA disproportionately affects females more than males, with 62% of those with OA being female [6]. Meanwhile, it is important to note that in populations less than 45 years of age, OA has been found to be more common in males than females. In populations older than 45 years of age, it is more common in women as well. There is some speculation that it could be due to estrogen, which is found in women. The most common ethnicity suffering from OA is non-Hispanic white populations [8].

According to Cui et al., OA is a very serious and prevalent disease not only in the US but also worldwide [4]. Specifically, 242 million people internationally suffer from symptomatic osteoarthritis. It is estimated that the annual total cost of OA is $136 billion, with an average direct cost of $11,000 per person per year [9]. A third of people with OA have five or more other chronic conditions, such as hypertension and diabetes [9]. OA has been widely noted to negatively affect sleep quality, mood, and quality of life, which will make it harder to manage those chronic conditions mentioned above. As a result, OA increases the risk of developing heart disease by 50% on average and a 55% increase in all-cause mortality [10]. This is important to see the vast impact that OA has had on both patients suffering from OA and the medical community.

As mentioned earlier, there are a number of risk factors associated with OA. Joint injury or overuse, such as repetitive microstress to the joint, can cause damage to the joint due to degenerative forces leading to OA by degrading the cartilage within the joint. Similar to diabetes, the older an individual gets, the more likely it is that they will develop OA. In terms of gender, women are at a higher risk for OA, especially at older ages [11]. Another key risk factor that one must keep in mind, similar to diabetes, is obesity [12]. Individuals with extra weight have added stress on their joints, particularly those "weight-bearing joints" such as the hips and knees. This stress increases the risk of OA through overuse and strain on those joints. In addition, individuals with family members who have been diagnosed with OA are more likely to develop OA due to genetic mutations and family history traits such as obesity. Especially hand and knee OA are seen in higher numbers within the realm of genetics [7,12]. As stated previously, while non-Hispanic whites have the highest percentages of OA, Asians are noted to have the least risk for OA [7,12].

Although there is no universal standard to treat or diagnose OA, there are various reviews of symptoms, physical examinations, imaging, and laboratory testing that one can perform. A rheumatologist, a physician in particular who specializes in arthritis, conducts a systematic review. OA is also treated with a combination of lifestyle alterations and supportive treatments. Those with OA are recommended to increase physical activity as tolerated, participate in weight loss programs, and use pain relief and anti-inflammatory medications. Supportive devices such as crutches are recommended to avoid additional strain on joints. Joint injections with steroids and other regenerative therapies are next, as is joint replacement surgery if other treatment options have not been effective.

Methods

Studies selected for this review article utilized PubMed as a search engine to compile the information, and a general PubMed search was completed with the incidence of diabetes mellitus (diabetes) and OA. In addition to PubMed, the CDC website was used to find accurate and up-to-date statistics, and an excerpt from a book was also utilized. Before the screening, duplicate studies were removed. Data from 2015-2022 was assessed for relevance; only reviews performed in the past eight years and performed in the US were accepted in the primary search. The large number of results was further refined by looking at manuscripts that focused specifically on relevant risk factors such as obesity, joint mobility issues, etc. When a number of risk factors kept recurring in a similar manner, those manuscripts were then selected to focus on. This information was kept track of in an Excel sheet in order to compare and contrast the similarities between diabetes and osteoarthritis.

Second, there was a separate search done broadly to determine the overlap between diabetes and osteoarthritis. In databases such as PubMed, the following keywords were used to refine the search criteria: OA, diabetes, risk factors, obesity, and inflammation. The resulting studies were further narrowed by looking at only those in the past eight years or less (2015 onwards). Upon looking into the specific mechanisms of diabetes and OA, certain topics were repeated, such as IL-1 and damage due to oxidative stress.

**Figure 1:**
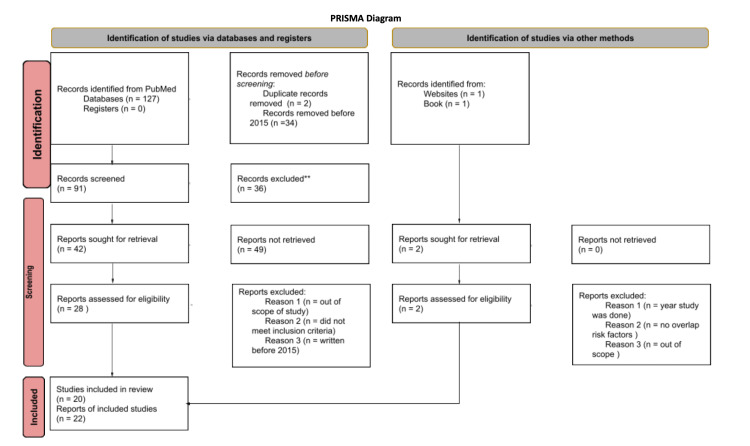
PRISMA diagram detailing methods process. Figure original © Harshita Nadella adopted from the PRISMA 2020 statement.

Lastly, there was a search done with specific overlaps, such as the role of IL-1b in both diabetes and OA, with specific keywords such as interleukin-1 (IL-1), tumor necrosis factor (TNF) alpha, and oxidative stress. This yielded the most specific pathogenicity and molecular connection between these two chronic diseases. This overlap provided the information for the assessment to gain insight into the connection between OA and diabetes. This process of including references was tracked using the PRISMA diagram (Figure 1).

## Review

Results: Possible overlap

Twenty-two studies were included in the final review, and the results are discussed below. The most frequent co-morbid disease in individuals with diabetes (types I and II) is OA [11]. The main purpose of this paper is to further develop the link that exists on a microbiological level between diabetes and OA. Both diabetes and osteoarthritis are chronic diseases, and they both have very similar risk factors such as obesity and age. In a large percentage of the population, such as those with the risk factors discussed above, both diseases coexist. The increased risk of OA and diabetes with aging and obesity is multifaceted. One of the main hypothesized reasons is that with age comes a decline in cell function and a decrease in telomere length [12]. Aging is associated with diabetes because beta cells' function in the pancreas declines as we get older. Similarly, in OA, older chondrocytes are "more likely than young chondrocytes" to secrete inflammatory mediators [13]. As a result, those inflammatory mediators, such as IL-1b, are directly related to cartilage degradation because of the oxidative stress produced. Obesity and age in conjunction lead to a higher load placed on joints, which can add to the cartilage damage found in OA. Aging is directly due to a "decline in mitochondrial health," which has been hypothesized to contribute to both diabetes and cartilage degradation and thus OA [14]. The function of mitochondria is to generate ATP (adenosine triphosphate) and carry oxygen, both of which play vital roles in diabetes and OA.

While diabetes is associated with hyperlipidemia and alterations in lipid metabolism are well established, there is also a relationship between lipid metabolism and OA. There is emerging evidence that demonstrates a direct connection between "alterations in lipid metabolism and hyperglycemia with cartilage health and subchondral bone" [15]. This clearly contributes to the development and progression of OA. Individuals with arthritis have a 61% higher risk of developing diabetes compared to those without a joint disease [16]. That is likely attributable to the fact that both diseases are characterized by disturbances in "cellular metabolism," which are caused by the alterations in lipid metabolism mentioned above [17]. Those disturbances are due to a transient insulin-resistant state that is formed due to chronic inflammation, which is common in both diabetics and OA [18].

As mentioned above, hyperglycemia can lead to negative impacts on the subchondral bone, which highlights the idea that diabetes itself is a risk factor for OA. Obesity and a number of metabolic syndromes, such as diabetes, have been described as independent risk factors for OA [19]. Insulin resistance might also impair joint tissue due to "local insulin resistance of the diabetic synovial membrane" [15,19]. Due to this transient insulin-resistant state, there is systemic low-grade inflammation, which involves cytokines such as IL-1, IL-6, and TNF alpha, leading to OA [15,19]. In a study using animal models of rodents, diabetic rodents displayed more "spontaneous and more severe experimental OA" in comparison with their non-diabetic rodents [13,19]. This negative impact of diabetes could be explained by the induction of "oxidative stress and pro-inflammatory cytokines." These cytokines also increase with age [12,19].

There are two very prevalent end products of metabolism that are present in diabetic patients: advanced glycation end products (AGEs) and diacylglycerol (DAG). AGEs are proteins or lipids that become glycated after exposure to sugar. They form when excess protein or fat in the bloodstream combines with sugar in a glycation process. DAG is a diacylglycerol that is generated in the process of food digestion. Both of these glucose derivatives are highly elevated in not only diabetic patients but also patients with OA [19].

This is because they are involved in the activation of inflammatory processes, which are highly associated with OA. The possible link between diabetes and OA can be due to this inflammatory process, end products, and increased expression of pro-inflammatory cytokines. Hyperglycemia and low-grade stress placed on synovial joints both lead to increased inflammation through pro-inflammatory cytokines such as IL-1, IL-6, and TNF-alpha [20]. That inflammatory process results in increased production of end products such as those in Table 2. Thus, this sheds light on the interconnectedness of these two chronic illnesses.

**Table 2:** This table demonstrates the cellular role present in the pancreatic beta cells involving mitochondrial reactive oxygen species generation with AGE and DAG [3]. Figure original © Harshita Nadella.

ROS (reactive oxygen species)	Function
NADH (nicotinamide adenine dinucleotide)	Provides electrons for aerobic ATP production
FADH2 (flavin adenine dinucleotide)	High energy electron carrier, transports electrons produced in glycolysis and Krebs cycle
AGE	Production of Maillard reaction, proteins or lipids that become glycated after exposure to sugar contributing to development of atherosclerosis
DAG	Secondary lipid messenger for transducing signals downstream of many receptors for activation, proliferation, migration

It is evident that diabetes and OA both coexist. It is important to note, however, that type 2 diabetes can have a pathological effect on OA through two major pathways: oxidative stress and low-grade chronic inflammation, which lead to chronic hyperglycemia, and insulin resistance, which are mentioned above and lead negatively to OA (Table 3).

**Table 3:** This table demonstrates the negative effect of an individual’s body from free radical oxidative stress. As one can gather both chronic inflammation leading to OA and diabetes are listed in this figure [21].  Figure original © Harshita Nadella.

Effect of free radical stress	Organ system
Brain	Alzheimer’s disease, Parkinson’s disease, autism spectrum disorder, migraine, depression, dementia, bipolar disorder, cancer
Eyes	Macular degeneration, retinal degeneration, cataracts
Heart	Myocardial Infarction, hypertension, stroke, atherosclerosis, angina, diabetes
Kidney	Chronic kidney disease, renal nephritis
Skin	Wrinkles, acne, eczema, psoriasis, dermatitis, cancer
Joints	Arthritis
Immune system	Chronic inflammation, HIV, herpes, Crohn’s disease, hepatitis, lupus, cancer
Blood vessels	Atherosclerosis, hypertension, varicose veins, hyperlipidemia
Lung	Asthma, COPD, allergies, cancer, bronchitis
Eyes	Macular degeneration, retinal degeneration, cataracts

There are specific interleukins tied to inflammation that are also associated with both diabetes and OA. Dr. Steve Abramson, MD, of New York University (NYU) in New York City, identified genetic associations with IL1α, IL1β, IL1RN, and COX2, which have the potential to lead to a number of inflammatory pathways associated with knee OA. Specifically, these research efforts have demonstrated that changes in gene expression result in increased production of inflammatory cytokines and matrix-degrading enzymes [6,20]. In addition, IL-1-beta and TNF-α are noted to "induce chondrocytes to produce proteases, chemokines, nitric oxide, and eicosanoids" such as prostaglandins and leukotrienes [21]. That same IL-1B is a target for many drugs tied to type 2 diabetes to "improve insulin secretion and action and glycemic control" [21]. This is because it is key in linking obesity and dyslipidemia, which can help explain cardiovascular complications found in a myriad of diabetic patients. Some interleukins, including those mentioned above, are known to further produce inflammatory cytokines in order to drive catabolic pathways, inhibit matrix synthesis, and promote cellular apoptosis [6,21]. Thus, interleukins play a major role in both diabetes and OA.

Furthermore, reactive oxygen species (ROS) likely play a key role in the inflammatory process and cellular apoptosis associated with OA and diabetes (Table 2). ROS are types of molecules that contain oxygen and easily react with other molecules in a cell, proving to be harmful to that cell. Superoxide anions, such as hydrogen peroxide, hydroxyl radicals, and nitric oxide, directly "promote chondrocyte apoptosis," most likely by negatively affecting mitochondrial function and disrupting the flow of oxygen [13,21]. As a result, many new potential drugs have been studied to observe the "efficacy of targeting IL-1b" in controlling both OA and diabetes [19,21]. Furthermore, as discussed above, Il-1b plays a significant role in the formation of atherosclerosis, which shows its importance as it leads to cardiovascular events [17,22]. The evidence discussed above does highlight the fact that interleukin therapy can prove to be especially beneficial in obese patients, which the US has seen a stark rise in since the COVID-19 pandemic.

**Figure 2:**
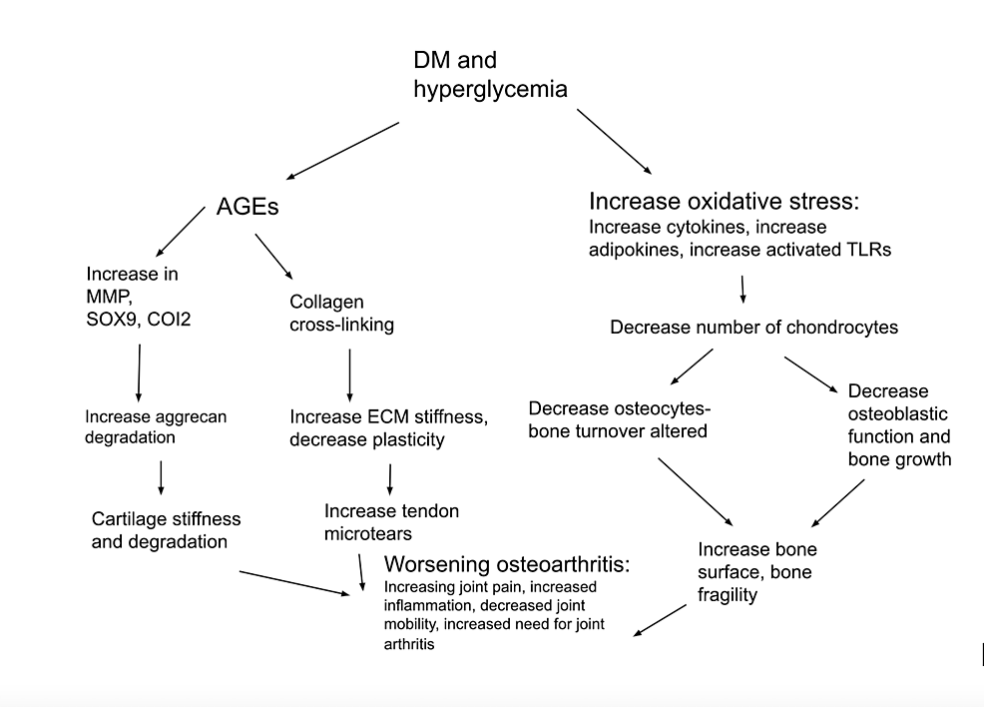
This figures outlines the connection between diabetes and osteoarthritis described in the paper. Figure original © Harshita Nadella.

New evidence has also been shed on the idea that IL-10 plays a role in the interrelationship between OA and diabetes [18,22]. In contrast with IL-1b, which is pro-inflammatory, IL-10 has robust anti-inflammatory properties that play a central role in limiting the body’s immune response and preventing damage, thus maintaining its homeostasis. As a result, IL-10 might have a protective role in both OA and type 2 diabetes. It is hypothesized that there is decreased availability of the anti-inflammatory IL-10 in both OA and diabetes [6,22]. As mentioned previously, the hypothesis that both OA and diabetes are based on imbalanced molecular pathways is further strengthened "with the putative crucial role of anti-inflammatory cytokines" such as Il-10 [11,22]. The chronic inflammation pathways are hyperactive in both chronic diseases. This information could be used to develop new therapeutic strategies that aim to increase the levels of IL-10 in those suffering from chronic diseases associated with inflammation. All the information above has been outlined in Figure 2.

## Conclusions

There is a very large overlap between OA and diabetes. A number of similar risk factors, including obesity and age, lead to the onset of both conditions. In addition, there are a plethora of overlap mechanisms, such as a decline in cell function, mitochondrial dysfunction, and dysregulation in lipid metabolism, which can lead to hyperlipidemia, which is a risk factor for both OA and diabetes. One of the most important mechanisms associated with both conditions is the chronic inflammation and cellular dysfunction of specific interleukins, such as IL-1b. Pro-inflammatory cytokines, including IL-1b and TNFa, have been identified in increasing numbers in both conditions, especially in obese patients. More research and associated funding are needed to identify anti-inflammatory pathways that can be used for those millions suffering from both OA and diabetes.

Patients who are suffering from atherosclerosis (CAD) and obesity are found to have more inflammatory tissue as well. Adipokines have also been found to have a direct relationship with Il-1b and an indirect relationship with Il-10. As new therapeutic approaches are being made for OA and diabetes, those same approaches can be used for addressing obesity as a secondary approach after lifestyle methods. All in all, it is vital that we recognize the interrelationship between OA and diabetes in order to better develop treatments in the future.
